# Crystal structure of bis­[4-(di­methyl­amino)­pyridine-κ*N* ^1^]bis­(methanol-κ*O*)bis­(thio­cyanato-κ*N*)manganese(II)

**DOI:** 10.1107/S2056989015007318

**Published:** 2015-04-30

**Authors:** Stefan Suckert, Inke Jess, Christian Näther

**Affiliations:** a Institut für Anorganische Chemie, Christian-Albrechts-Universität Kiel, Max-Eyth-Strasse 2, 24118 Kiel, Germany

**Keywords:** crystal structure, discrete complex, octa­hedral coordination, hydrogen bonding

## Abstract

The whole mol­ecule of the title compound, [Mn(NCS)_2_(CH_3_OH)_2_(C_5_H_6_N_2_)_2_], is generated by inversion symmetry. The Mn^II^ ion, which is located on an inversion center, is coordinated by two 4-(di­methyl­amino)­pyridine ligands, two methanol ligands and two terminally *N*-bonded thio­cyanate anions, forming a slightly distorted octa­hedron. In the crystal, mol­ecules are linked by O—H⋯S hydrogen bonds, forming chains extending along the *a*-axis direction.

## Related literature   

For the structure of another discrete complex with 4-(di­methyl­amino)­pyridine and thio­cyanate ligands, see: Chen *et al.* (2007 ▸). For general background to this work, see: Näther *et al.* (2013 ▸).
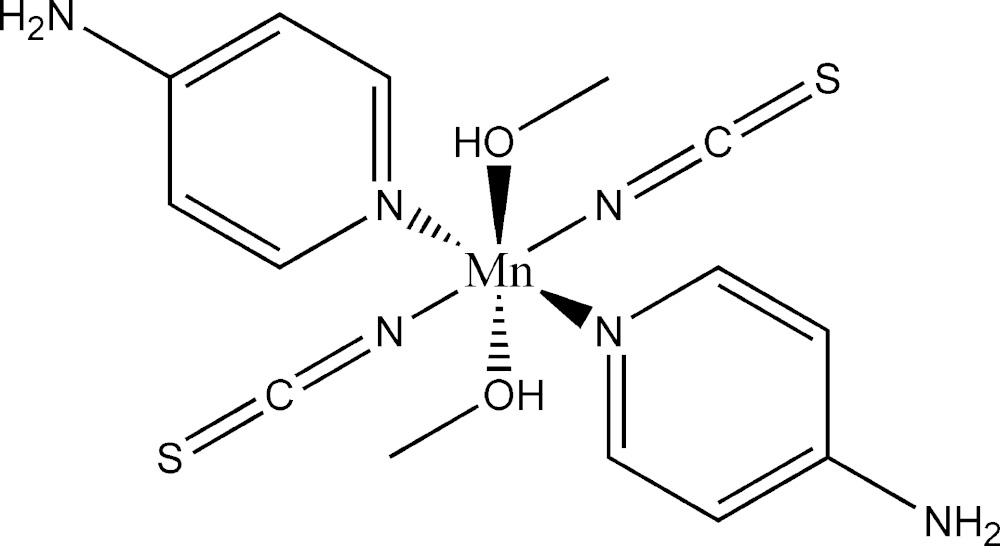


## Experimental   

### Crystal data  

[Mn(NCS)_2_(CH_4_O)_2_(C_5_H_6_N_2_)_2_]
*M* *_r_* = 479.52
Triclinic, 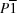

*a* = 7.0771 (7) Å
*b* = 8.1586 (8) Å
*c* = 10.6491 (10) Å
α = 76.381 (11)°
β = 81.672 (11)°
γ = 79.809 (11)°
*V* = 584.72 (10) Å^3^
*Z* = 1
Mo *K*α radiation
μ = 0.77 mm^−1^
*T* = 180 K
0.16 × 0.10 × 0.04 mm

### Data collection  

Stoe IPDS-1 diffractometer
Absorption correction: numerical (*X-SHAPE* and *X-RED32*; Stoe & Cie, 2008 ▸) *T*_min_ = 0.903, *T*_max_ = 0.959
4585 measured reflections
2459 independent reflections
1885 reflections with *I* > 2σ(*I*)
*R*_int_ = 0.039

### Refinement  

*R*[*F* ^2^ > 2σ(*F* ^2^)] = 0.038
*wR*(*F* ^2^) = 0.100
*S* = 1.04
2459 reflections
133 parameters
H-atom parameters constrained
Δρ_max_ = 0.30 e Å^−3^
Δρ_min_ = −0.59 e Å^−3^

### 

Data collection: *X-AREA* (Stoe & Cie, 2008 ▸); cell refinement: *X-AREA*; data reduction: *X-AREA*; program(s) used to solve structure: *SHELXS97* (Sheldrick, 2008 ▸); program(s) used to refine structure: *SHELXL2013* (Sheldrick, 2015 ▸); molecular graphics: *XP* in *SHELXTL* (Sheldrick, 2008 ▸) and *DIAMOND* (Brandenburg, 1999 ▸); software used to prepare material for publication: *publCIF* (Westrip, 2010 ▸).

## Supplementary Material

Crystal structure: contains datablock(s) I, global. DOI: 10.1107/S2056989015007318/sj5446sup1.cif

Structure factors: contains datablock(s) I. DOI: 10.1107/S2056989015007318/sj5446Isup2.hkl

**Fig. 1.**
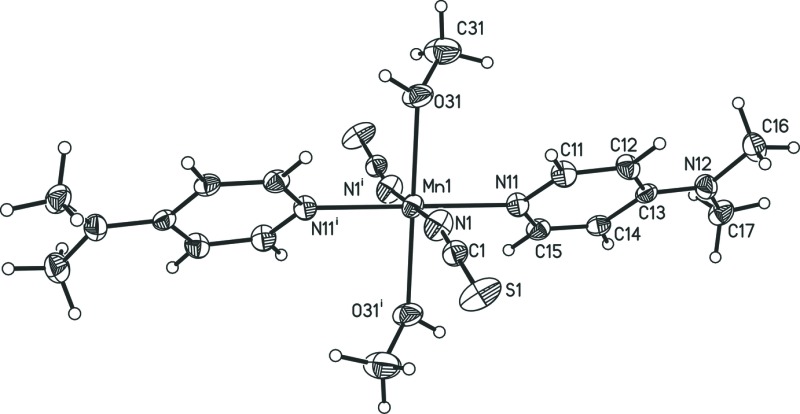
Structure of the title complex with atom labelling. Displacement ellipsoids are drawn at the 50% probability level. Symmetry code: i = -x+1,-y,-z+1.

**Fig. 2.**
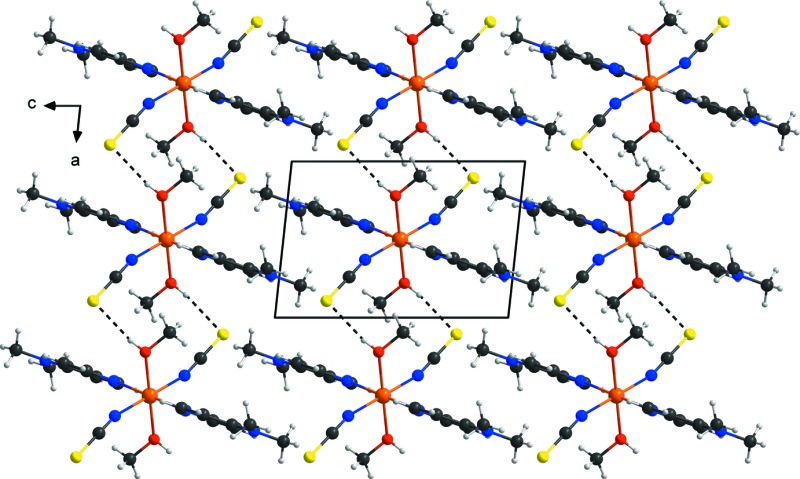
Crystal structure of the title compound viewed perpendicular to the crystallographic a,c plane.

. DOI: 10.1107/S2056989015007318/sj5446fig1.tif
Structure of the title complex with atom labelling. Displacement ellipsoids are drawn at the 50% probability level. Symmetry code: i = −x+1,-y,-z+1.

a,c . DOI: 10.1107/S2056989015007318/sj5446fig2.tif
Crystal structure of the title compound viewed perpendicular to the crystallographic *a,c* plane.

CCDC reference: 1059105

Additional supporting information:  crystallographic information; 3D view; checkCIF report

## Figures and Tables

**Table 1:** Hydrogen-bond geometry (, )

*D*H*A*	*D*H	H*A*	*D* *A*	*D*H*A*
O31H1*O*S1^i^	0.85	2.42	3.2409(18)	161

Symmetry code: (i) 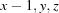.

## supplementary crystallographic information

### S1. Synthesis and crystallization

MnSO_4_.H_2_O was purchased from Merck and 4-(di­methyl­amino)­pyridine and Ba(NCS)_2_.3 H_2_O were purchased from Alfa Aesar. Mn(NCS)_2_ was synthesized by stirring 17.97 g (58.44 mmol) Ba(NCS)_2_.3 H_2_O and 9.88 g (58.44 mmol) MnSO_4_.H_2_O in 300 mL H_2_O at room temperature for three hours. The white precipitate of BaSO_4_ was filtered of and the solvent removed with a rotary evaporator. The homogeneity of the product was investigated by X-ray powder diffraction and elemental analysis. The title compound was prepared by the reaction of (0.18 mmol) 30.8 mg Mn(NCS)_2_ and (0.3 mmol) 36.7 mg 4-(di­methyl­amino)­pyridine in 1.0 mL methanol at room temperature. After a few days colorless plate shaped crystals of the title compound were obtained.

### S2. Refinement

The C—H H atoms were positioned with idealized geometry and were refined isotropically with *U*_iso_(H) = 1.2 *U*_eq_(C) (1.5 for methyl H atoms) using a riding model with C—H = 0.95 Å for aromatic and and C—H = 0.98 Å for methyl H atoms. The O—H H atom was located in a difference map, its bond length set to ideal values of 0.85 Å and refined with *U*_iso_(H) = 1.5 *U*_eq_(O)using a riding model.

### Crystal data

**Table:**

[Mn(NCS)_2_(CH_4_O)_2_(C_5_H_6_N_2_)_2_]	*Z* = 1
*M**_r_* = 479.52	*F*(000) = 251
Triclinic, *P*1	*D*_x_ = 1.362 Mg m^−^^3^
*a* = 7.0771 (7) Å	Mo *K*α radiation, λ = 0.71073 Å
*b* = 8.1586 (8) Å	Cell parameters from 4585 reflections
*c* = 10.6491 (10) Å	θ = 2.6–27.0°
α = 76.381 (11)°	µ = 0.77 mm^−^^1^
β = 81.672 (11)°	*T* = 180 K
γ = 79.809 (11)°	Plate, colorless
*V* = 584.72 (10) Å^3^	0.16 × 0.10 × 0.04 mm

### Data collection

**Table:**

Stoe IPDS-1 diffractometer	1885 reflections with *I* > 2σ(*I*)
Radiation source: fine-focus sealed tube	*R*_int_ = 0.039
phi scans	θ_max_ = 27.0°, θ_min_ = 2.6°
Absorption correction: numerical (*X-SHAPE* and *X-RED32*; Stoe & Cie, 2008)	*h* = −9→9
*T*_min_ = 0.903, *T*_max_ = 0.959	*k* = −10→10
4585 measured reflections	*l* = −13→13
2459 independent reflections	

### Refinement

**Table:**

Refinement on *F*^2^	0 restraints
Least-squares matrix: full	Hydrogen site location: mixed
*R*[*F*^2^ > 2σ(*F*^2^)] = 0.038	H-atom parameters constrained
*wR*(*F*^2^) = 0.100	*w* = 1/[σ^2^(*F*_o_^2^) + (0.0598*P*)^2^] where *P* = (*F*_o_^2^ + 2*F*_c_^2^)/3
*S* = 1.04	(Δ/σ)_max_ < 0.001
2459 reflections	Δρ_max_ = 0.30 e Å^−^^3^
133 parameters	Δρ_min_ = −0.59 e Å^−^^3^

### Special details

**Table:**

Geometry. All esds (except the esd in the dihedral angle between two l.s. planes) are estimated using the full covariance matrix. The cell esds are taken into account individually in the estimation of esds in distances, angles and torsion angles; correlations between esds in cell parameters are only used when they are defined by crystal symmetry. An approximate (isotropic) treatment of cell esds is used for estimating esds involving l.s. planes.

### Fractional atomic coordinates and isotropic or equivalent isotropic displacement parameters (Å^2^)

**Table:**

	*x*	*y*	*z*	*U*_iso_*/*U*_eq_	
Mn1	0.5000	0.0000	0.5000	0.02111 (15)	
N1	0.6294 (3)	0.0926 (3)	0.63997 (19)	0.0312 (5)	
C1	0.7395 (3)	0.1199 (3)	0.7004 (2)	0.0236 (5)	
S1	0.89299 (9)	0.15954 (10)	0.78630 (6)	0.0394 (2)	
O31	0.2205 (2)	0.1737 (2)	0.53900 (16)	0.0339 (4)	
H1O	0.1462	0.1444	0.6079	0.051*	
C31	0.0945 (4)	0.2643 (4)	0.4439 (3)	0.0486 (7)	
H31A	−0.0136	0.3337	0.4847	0.073*	
H31B	0.0448	0.1828	0.4074	0.073*	
H31C	0.1659	0.3386	0.3743	0.073*	
N11	0.5945 (3)	0.2090 (2)	0.33954 (17)	0.0222 (4)	
N12	0.7263 (3)	0.6192 (2)	0.03652 (18)	0.0274 (4)	
C11	0.5795 (3)	0.3682 (3)	0.3576 (2)	0.0264 (5)	
H11	0.5369	0.3866	0.4427	0.032*	
C12	0.6210 (3)	0.5063 (3)	0.2624 (2)	0.0252 (5)	
H12	0.6057	0.6157	0.2825	0.030*	
C13	0.6867 (3)	0.4861 (3)	0.1345 (2)	0.0210 (4)	
C14	0.7077 (3)	0.3182 (3)	0.1162 (2)	0.0230 (4)	
H14	0.7554	0.2942	0.0334	0.028*	
C15	0.6594 (3)	0.1895 (3)	0.2177 (2)	0.0235 (5)	
H15	0.6727	0.0783	0.2011	0.028*	
C16	0.6858 (4)	0.7919 (3)	0.0569 (3)	0.0353 (6)	
H16A	0.7210	0.8713	−0.0243	0.053*	
H16B	0.7612	0.8015	0.1244	0.053*	
H16C	0.5479	0.8196	0.0844	0.053*	
C17	0.7938 (3)	0.5957 (3)	−0.0944 (2)	0.0309 (5)	
H17A	0.8140	0.7059	−0.1514	0.046*	
H17B	0.6972	0.5482	−0.1267	0.046*	
H17C	0.9158	0.5171	−0.0934	0.046*	

### Atomic displacement parameters (Å^2^)

**Table:**

	*U*^11^	*U*^22^	*U*^33^	*U*^12^	*U*^13^	*U*^23^
Mn1	0.0216 (2)	0.0237 (3)	0.0185 (2)	−0.00575 (18)	0.00127 (17)	−0.00564 (18)
N1	0.0330 (10)	0.0364 (12)	0.0282 (10)	−0.0112 (9)	−0.0016 (8)	−0.0117 (9)
C1	0.0254 (11)	0.0242 (11)	0.0201 (10)	−0.0035 (8)	0.0040 (8)	−0.0067 (9)
S1	0.0261 (3)	0.0703 (5)	0.0288 (3)	−0.0110 (3)	0.0005 (2)	−0.0240 (3)
O31	0.0250 (8)	0.0444 (11)	0.0258 (8)	0.0030 (7)	0.0033 (6)	−0.0046 (8)
C31	0.0373 (15)	0.062 (2)	0.0343 (14)	0.0113 (13)	−0.0022 (11)	−0.0017 (13)
N11	0.0272 (9)	0.0210 (9)	0.0188 (9)	−0.0037 (7)	0.0014 (7)	−0.0070 (7)
N12	0.0300 (10)	0.0241 (10)	0.0248 (10)	−0.0039 (8)	0.0015 (8)	−0.0017 (8)
C11	0.0325 (12)	0.0263 (12)	0.0210 (11)	−0.0043 (9)	0.0028 (8)	−0.0100 (9)
C12	0.0306 (11)	0.0201 (11)	0.0267 (11)	−0.0032 (9)	−0.0011 (9)	−0.0100 (9)
C13	0.0143 (9)	0.0245 (11)	0.0238 (10)	−0.0031 (8)	−0.0019 (7)	−0.0040 (9)
C14	0.0205 (10)	0.0270 (11)	0.0210 (10)	−0.0009 (8)	0.0022 (8)	−0.0090 (9)
C15	0.0222 (10)	0.0242 (11)	0.0258 (11)	−0.0021 (8)	0.0013 (8)	−0.0119 (9)
C16	0.0405 (14)	0.0234 (12)	0.0399 (14)	−0.0064 (10)	−0.0011 (11)	−0.0034 (11)
C17	0.0274 (11)	0.0402 (14)	0.0217 (11)	−0.0070 (10)	−0.0001 (9)	0.0001 (10)

### Geometric parameters (Å, º)

**Table:**

Mn1—N1^i^	2.192 (2)	N12—C16	1.448 (3)
Mn1—N1	2.192 (2)	N12—C17	1.452 (3)
Mn1—N11	2.2302 (17)	C11—C12	1.370 (3)
Mn1—N11^i^	2.2302 (17)	C11—H11	0.9500
Mn1—O31	2.2676 (17)	C12—C13	1.412 (3)
Mn1—O31^i^	2.2676 (17)	C12—H12	0.9500
N1—C1	1.160 (3)	C13—C14	1.408 (3)
C1—S1	1.634 (2)	C14—C15	1.368 (3)
O31—C31	1.429 (3)	C14—H14	0.9500
O31—H1O	0.8500	C15—H15	0.9500
C31—H31A	0.9800	C16—H16A	0.9800
C31—H31B	0.9800	C16—H16B	0.9800
C31—H31C	0.9800	C16—H16C	0.9800
N11—C11	1.341 (3)	C17—H17A	0.9800
N11—C15	1.349 (3)	C17—H17B	0.9800
N12—C13	1.355 (3)	C17—H17C	0.9800
			
N1^i^—Mn1—N1	180.0	C13—N12—C17	121.3 (2)
N1^i^—Mn1—N11	89.19 (7)	C16—N12—C17	117.81 (19)
N1—Mn1—N11	90.81 (7)	N11—C11—C12	124.8 (2)
N1^i^—Mn1—N11^i^	90.81 (7)	N11—C11—H11	117.6
N1—Mn1—N11^i^	89.19 (7)	C12—C11—H11	117.6
N11—Mn1—N11^i^	180.0	C11—C12—C13	120.0 (2)
N1^i^—Mn1—O31	90.50 (7)	C11—C12—H12	120.0
N1—Mn1—O31	89.50 (7)	C13—C12—H12	120.0
N11—Mn1—O31	89.07 (6)	N12—C13—C14	122.6 (2)
N11^i^—Mn1—O31	90.93 (6)	N12—C13—C12	122.2 (2)
N1^i^—Mn1—O31^i^	89.50 (7)	C14—C13—C12	115.22 (19)
N1—Mn1—O31^i^	90.50 (7)	C15—C14—C13	120.1 (2)
N11—Mn1—O31^i^	90.93 (6)	C15—C14—H14	120.0
N11^i^—Mn1—O31^i^	89.07 (6)	C13—C14—H14	120.0
O31—Mn1—O31^i^	180.0	N11—C15—C14	124.7 (2)
C1—N1—Mn1	162.79 (19)	N11—C15—H15	117.6
N1—C1—S1	179.4 (2)	C14—C15—H15	117.6
C31—O31—Mn1	125.44 (16)	N12—C16—H16A	109.5
C31—O31—H1O	105.1	N12—C16—H16B	109.5
Mn1—O31—H1O	119.1	H16A—C16—H16B	109.5
O31—C31—H31A	109.5	N12—C16—H16C	109.5
O31—C31—H31B	109.5	H16A—C16—H16C	109.5
H31A—C31—H31B	109.5	H16B—C16—H16C	109.5
O31—C31—H31C	109.5	N12—C17—H17A	109.5
H31A—C31—H31C	109.5	N12—C17—H17B	109.5
H31B—C31—H31C	109.5	H17A—C17—H17B	109.5
C11—N11—C15	115.11 (18)	N12—C17—H17C	109.5
C11—N11—Mn1	120.90 (14)	H17A—C17—H17C	109.5
C15—N11—Mn1	123.89 (15)	H17B—C17—H17C	109.5
C13—N12—C16	120.6 (2)		

Symmetry code: (i) −*x*+1, −*y*, −*z*+1.

### Hydrogen-bond geometry (Å, º)

**Table:**

*D*—H···*A*	*D*—H	H···*A*	*D*···*A*	*D*—H···*A*
O31—H1*O*···S1^ii^	0.85	2.42	3.2409 (18)	161

Symmetry code: (ii) *x*−1, *y*, *z*.
