# Supplementary figures and images for: Crystal structure of bis­[4-(di­methyl­amino)­pyridine-κN 1]bis­(methanol-κO)bis­(thio­cyanato-κN)manganese(II)
Source: Acta Crystallogr E Crystallogr Commun. 2015 Apr 30;71(Pt 5):m126. doi: 10.1107/S2056989015007318 (PMC4420091; doi:10.1107/S2056989015007318)

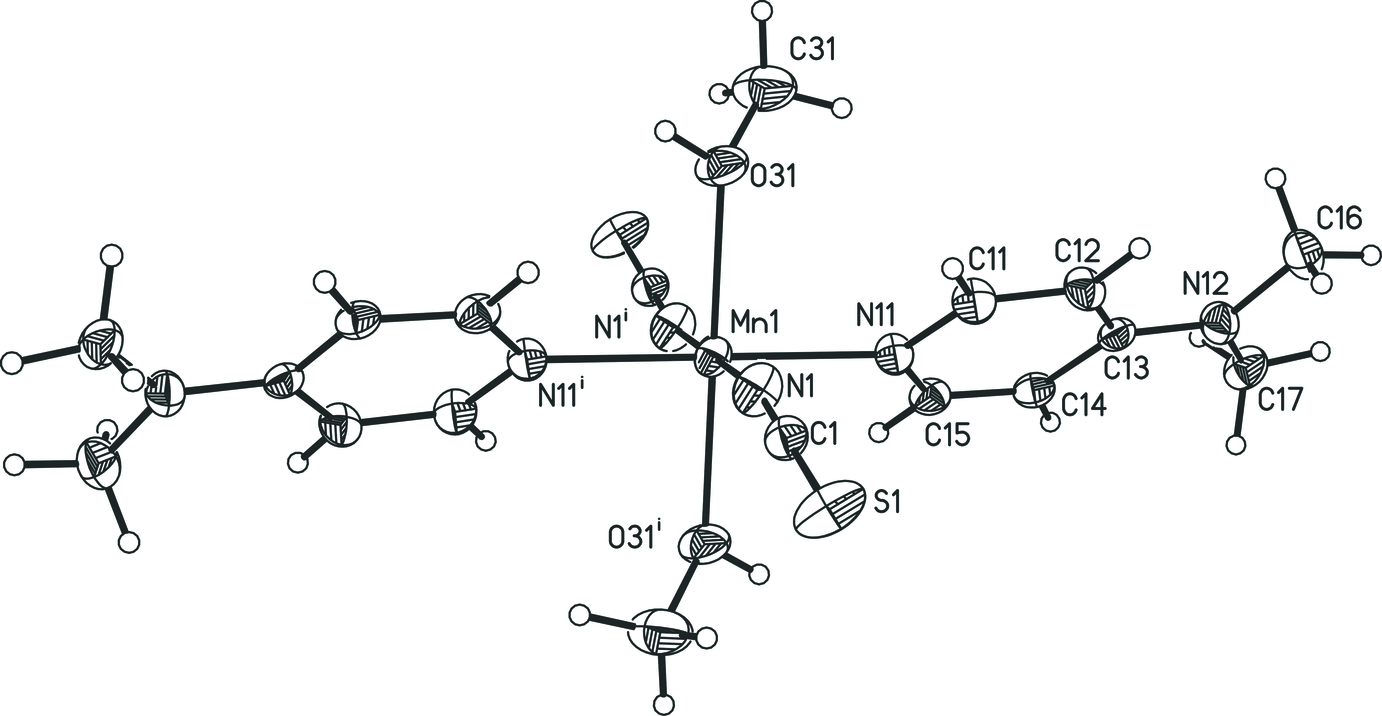

Supplement: Supplementary file 3 [file e-71-0m126-fig1.tif]

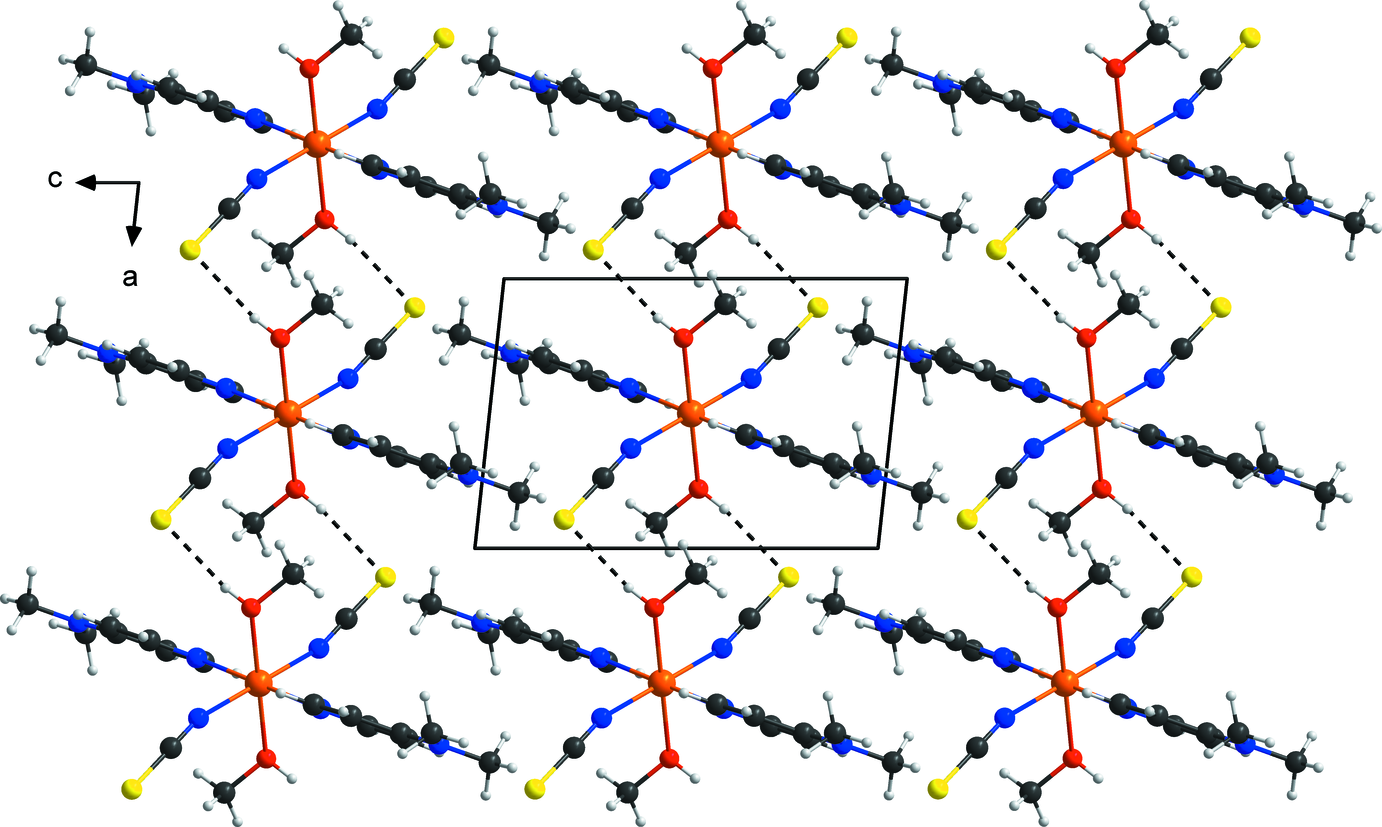

Supplement: Supplementary file 4 [file e-71-0m126-fig2.tif]
